# A Nanopore-Gated Subattoliter Silicon Nanocavity for Single-Molecule Trapping and Analysis without Applying an External Force

**DOI:** 10.1021/acsnano.6c06884

**Published:** 2026-06-22

**Authors:** Funing Liu, Qitao Hu, Anton Sabantsev, Giovanni Di Muccio, Shuangshuang Zeng, Mauro Chinappi, Sebastian Deindl, Zhen Zhang

**Affiliations:** † Division of Solid-State Electronics, Department of Electrical Engineering, 8097 Uppsala University, Uppsala SE-75121, Sweden; ‡ Department of Cell and Molecular Biology, Science for Life Laboratory, Uppsala University, Uppsala SE-75237, Sweden; § NY-Masbic, Department of Life and Environmental Sciences, 9294 Università Politecnica delle Marche,Via Brecce Bianche, Ancona 60131, Italy; ∥ National Future Biodiversity Center (NFBC), Palermo 90133, Italy; ⊥ Department of Industrial Engineering, University of Rome Tor Vergata, Roma 00133, Italy; # Interfaculty Institute of Biochemistry (IFIB), University of Tübingen, Tübingen 72074, Germany

**Keywords:** nanopore-gated nanocavity, subattoliter, single-molecule analysis, external force, nucleosome, molecular dynamics, weak interactions

## Abstract

Biomolecules exhibit dynamic conformations critical to their functions, yet observing these processes at the single-molecule level under native conditions remains a formidable challenge. While surface immobilization has been widely used to extend observation times, it can disrupt molecular dynamics and impede biological function. Recent advancements in single-molecule trapping techniques have addressed some limitations, but achieving precise, controllable, long-term trapping in a molecularly crowded environment without external forces remains difficult. Here, we introduce a nanopore-gated subattoliter silicon nanocavity that enables precise entropic trapping of individual biomolecules for extended observation times, eliminating the need for surface immobilization or external forces. Using nucleosomes as model systems, we demonstrate single-molecule Förster resonance energy transfer (smFRET) to monitor relative distances. With smFRET, we directly observe dynamic unwrapping and rewrapping events induced by the chromatin remodeling enzyme Chd1, as well as weak interactions between two nucleosomes trapped inside the nanocavity. Our data further demonstrate that an applied electric field can modulate the conformational properties of the macromolecules, emphasizing a key advantage of our device: it does not require an electric field to retain trapped molecules. We envision this nanocavity platform as a powerful tool for the interrogation of molecular dynamics without applying an external force in physiologically relevant environments, offering access to weak and transient interactions that are central to biological regulation.

## Graphical abstract

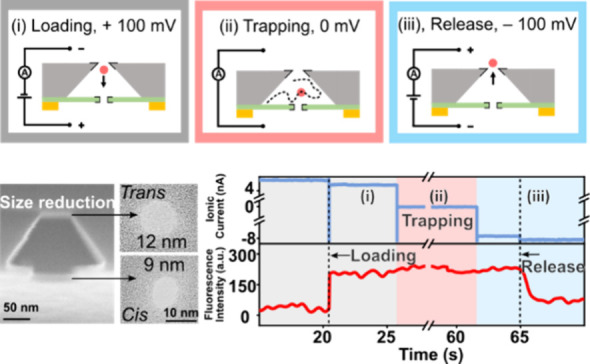

Biomolecules adopt diverse conformations essential for their functions. Structural biology reveals these states through high-resolution techniques like cryo-EM, which captures distinct conformations in macromolecular ensembles. While static snapshots reveal equilibrium states, they fail to capture the dynamics of state interconversion. In ensemble measurements, these transitions are often obscured due to asynchronous molecular behavior. Single-molecule techniques have revolutionized the study of complex biomolecular processes, enabling the observation of dynamic conformational changes and transient interactions that are often masked in ensemble measurements.^1–19^ A central challenge remains the need to observe single molecules over extended periods under conditions that preserve their native behavior.^20–25^

A widely used approach for extending observation times is to anchor biomolecules to a surface, such as a coverslip.^26^ Similarly, optical tweezers can trap single molecules by holding a bead, conjugated to the molecule, in place with a highly focused laser beam.^27^ While surface-immobilization-based methods have unquestionably yielded highly significant results and continue to evolve,^28,29^ the immobilization process can disrupt the molecule’s natural dynamics and, in some cases, impede its biological function.^30–32^ To overcome these limitations, several techniques have been developed in recent years for the longer-term observation of single molecules without immobilization. Methods such as the nanopore electro-osmotic trap (NEOtrap)^33^ utilize electro-osmotic forces to trap single molecules exploiting a DNA-origami nanosphere docked on a nanopore, allowing observation for hours. Similarly, the electrokinetic nanovalve relies on dielectrophoretic forces in lab-on-chip setups to guide and confine molecules,^34^ while the anti-Brownian electrophoretic trap (ABEL trap) uses real-time feedback voltage to counteract Brownian motion, enabling trapping in open environments.^23,24,35–38^ However, these elegant approaches depend on external electric fields, which could potentially perturb the molecule’s native dynamics.^39–41^ Electrostatic fluid traps^42–44^ and porous vesicle encapsulation techniques^45,46^ allow passive trapping of single molecules without the application of external forces. Despite their simplicity, these methods rely on stochastic loading, making precise control difficult and limiting their applicability to systems requiring selective or repeatable trapping. Entropic cages offer a promising alternative, confining single molecules through spatial constraints without external forces,^47–49^ differing from conventional nanopores generally report on molecules during translocation or while they are confined in the pore under an electric field.^50–52^ Although this approach is gentle and highly controllable, it is currently limited to large biomolecules, such as very long DNA molecules, due to the size of the apertures and cages. This limits its use for smaller biomolecules and complexes.

Despite these significant advancements, achieving a solution that fulfils all desired capabilities—controllable trapping of single molecules for extended observation times in the absence of the external force in a molecularly crowded environment^53,54^ (similar to the intracellular space, which often features high molecular concentrations) – remains a challenge. Here, we present a novel nanopore-gated subattoliter silicon nanocavity device as a robust entropic trapping platform for single-molecule trapping and analysis without applying any external force. Fabricated in a thin silicon membrane of a silicon-on-insulator (SOI) wafer using high-precision silicon processing, the nanocavity connects two liquid reservoirs through the *trans/cis* nanopore gates of different sizes, enabling ionic current flow under an applied electrical bias. The larger nanopore allows controlled electrical loading or release of single molecules between the nanocavity and one reservoir, while the smaller nanopore prevents unintended molecule escape. Real-time monitoring of ionic current can provide feedback for precise molecule loading. Once loaded, the electrical bias is switched off, and the molecule is entropically trapped for analysis. To fine-tune nanopore sizes for specific molecules, we developed a customized carbon deposition technique. Using nanopores of 12 and 9 nm, we demonstrated the controlled loading, release, and trapping of single or multiple 11 nm-sized nucleosomes. Furthermore, single-molecule Förster resonance energy transfer (smFRET)^55–57^ analysis within the nanodevice reveals the dynamics of trapped nucleosomes and highlights the modulatory effects of applied electric fields. We also used the device to directly observe weak interactions between two nucleosomes cotrapped in the nanocavity, demonstrating its ability to trap biomolecules precisely without applying any external force for extended single-molecule observation in a molecularly crowded environment.

## Results and Discussion

### Nanocavity Device Design and Operation

To enable controllable trapping of individual molecules, we designed a nanocavity device comprising a top silicon nanopore (*trans*), a subattoliter silicon nanocavity, and a bottom silicon nitride (SiN_*x*_) nanopore (*cis*). We fabricated the device in a free-standing silicon membrane of a silicon-on-insulator (SOI) wafer, with membrane and SiN_*x*_ layers 88 and 30 nm thick, respectively (Figures 1a, S1). To reduce background signal, we deposited a 50 nm-thick gold layer onto the SiN_*x*_ layer, 1 μm beyond the SiN_*x*_ nanopore. Adjusting membrane thickness and wet-etching duration^58^ allowed precise control over the nanocavity size. Moreover, we developed a carbon deposition technique to precisely reduce nanopore sizes. To characterize the resulting nanocavity device, we used cross-sectional scanning electron microscopy (SEM) before carbon deposition and transmission electron microscopy (TEM) after carbon deposition, revealing a 12 nm silicon nanopore and a 9 nm SiN_*x*_ nanopore (Figure 1b). These dimensions were specifically chosen to accommodate the target molecule investigated in this work. In addition, the solid surfaces of the nanocavity were passivated with BSA prior to the loading of actual target molecules to prevent surface sticking.

**Figure 1:**
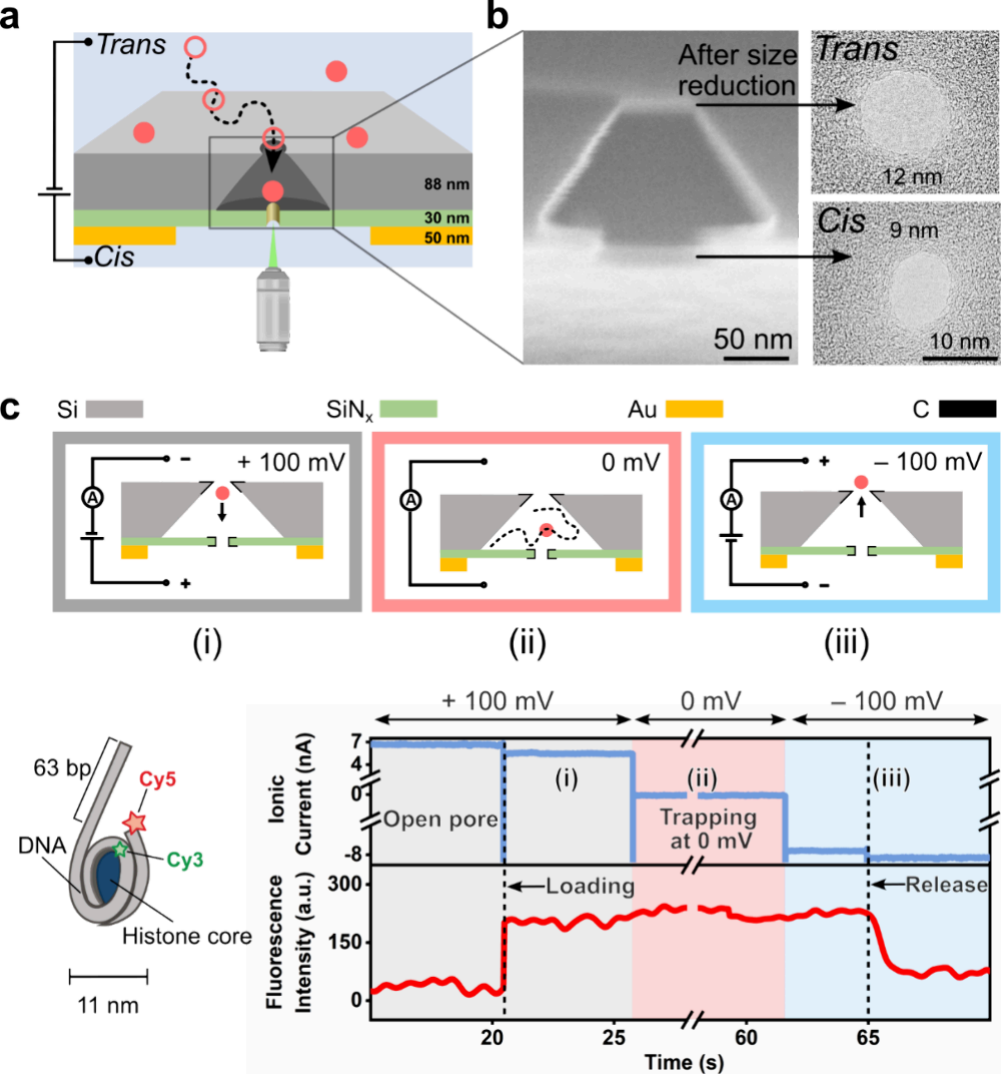
Nanocavity device structure, experimental setup, and working principle for the controllable loading, trapping, and release of a single molecule. (a) Schematic of the nanocavity device and the electro-optical setup, with a single-molecule fluorescence confocal microscope positioned beneath the *cis* side of the nanocavity. Upon application of an electric field, individual molecules are drawn into the nanocavity. (b) Cross-sectional SEM image of the nanocavity device prior to carbon deposition, and TEM images of the silicon pore on the *trans* side and the SiN_*x*_ pore on the *cis* side after carbon deposition for size reduction. (c) Upper: schematic representation of the controllable loading, trapping, and release of a single molecule. (i) Loading into the nanocavity at +100 mV. (ii) Trapping at 0 mV, with the dashed line indicating Brownian motion. (iii) Release upon voltage reversal to −100 mV. Lower: cartoon representation of the 11 nm fluorophore-labeled nucleosome. Time traces of ionic current (blue) and Cy5 fluorescence intensity (red), corresponding to the steps described in the schematic representation, for the loading experiments of 1 nM fluorophore-labeled nucleosomes in imaging buffer.

The thin membrane separates the imaging buffer containing electrolyte solution into *trans* and *cis* reservoirs (Figure 1a,b). The only fluid pathway connecting these reservoirs passes through the silicon nanopore, the nanocavity, and the SiN_*x*_ nanopore. Accordingly, applying a voltage between the reservoirs via Ag/AgCl electrodes is expected to generate an ionic current through this pathway. We reason that an electrical bias could pull molecules from the *trans* reservoir through the silicon nanopore into the nanocavity via electrophoretic^59^ or electroosmotic^60^ forces. A fluorescence microscope beneath the *cis* reservoir allows visualization of fluorophore-labeled molecules in the nanocavity (Figure 1a).

To assess the performance of our device using a biologically relevant macromolecular complex, we reconstituted mononucleosomes comprising histone octamers labeled with the FRET donor dye Cy3 on histone H2A­(K120C) and double-stranded DNA labeled with the acceptor dye Cy5 at the short linker end (Figure 1c). The nucleosome, the fundamental packaging unit of eukaryotic genomes, consists of a histone octamer wrapped by approximately 147 base pairs (bp) of DNA.^61^ Its positioning, modifications, and dynamics are critical for regulating genome accessibility and function.^62–66^

### Customizable Nanodevices with Tunable Nanopore Gate Sizes

To determine the optimal nanopore gate sizes for trapping FRET-labeled nucleosomes, we considered the following: if both the *trans* and *cis* nanopores were larger than the target molecule, the molecule would translocate through the nanocavity under electrical bias. If the *trans* nanopore were much larger and the *cis* nanopore smaller than the target molecule, the molecule would only be held within the nanocavity under electrical bias and could, upon bias removal, easily escape through the *trans* nanopore via Brownian motion. Conversely, if the *trans* nanopore could match the target molecule size and the *cis* nanopore were smaller, the molecule would be loaded into the nanocavity by electrical force and remain entropically trapped without an external field. Initial tests using 20 kb DNA under constant loading bias demonstrated dwell-time extension through nanopore size reduction, confirming that appropriately sized nanopores are critical for prolonged trapping (Devices a–c in Figure S2). Given the nucleosome diameter of ∼11 nm,^61^ we further refined nanopore dimensions using electron beam-induced carbon deposition (Figure S3). This method enabled the fabrication of several devices with defined I–V characteristics (Figure S4), including one with *trans* and *cis* nanopores of 12 and 9 nm (Device 1), respectively—dimensions ideally suited for trapping nucleosomes (Figure 1b).

To monitor loading, trapping, and release electrically and optically, we recorded ionic current and Cy5 acceptor fluorescence emission under Cy3 donor excitation with a 532 nm laser (Figure 1c). And ±100 mV biases were chosen in the following experiments to demonstrate proof-of-concept voltage-controlled loading and release. Upon application of a +100 mV loading bias to Device 1, we initially observed an open-pore baseline ionic current consistent with an empty nanocavity. A sudden decrease in ionic current by 1.1 nA signaled nucleosome loading into the nanocavity through the *trans* nanopore, whose size was chosen comparable to the largest nucleosome dimension of approximately 11 nm. Simultaneous Cy5 fluorescence increase confirmed the nucleosome loading. The smaller *cis* nanopore prevented translocation, effectively trapping the nucleosome within the nanocavity under bias. After removing bias, ionic current disappeared, however, Cy5 fluorescence remained, indicating entropic trapping for >30 s (Figures 1c, S5a,b). This trapping required no external force to maintain confinement (Figure S5d). Finally, reversing the bias voltage to −100 mV could force the release of the nucleosome from the nanocavity, as confirmed in Figures 1c and S5b, where both the magnitude of the ionic current and the fluorescence emission intensity return to the values near the baseline. In direct contrast, only translocation events were observed with Device 2 (Figure S5e,f), which had two larger nanopores (23 nm *trans*, 16 nm *cis*). Furthermore, a single nucleosome loaded into Device 3, featuring a larger 21 nm *trans* nanopore but smaller 10 nm *cis* nanopore, escaped immediately upon the removal of the electrical bias (Figure S5g,h). Repeat experiments show the same immediate escape of the loaded molecules upon removal of the bias in this device (Figure S5i), confirming that surface sticking of nucleosomes was prevented by BSA passivation. All subsequent experiments were performed with Device 1.

Interestingly, we did not observe the subsequent loading of a second nucleosome into Device 1 while maintaining a constant +100 mV loading bias, even after prolonged periods (Figure S5c). We reason that the first loaded molecule partially blocked the *cis* nanopore, causing the applied electrical bias to be redistributed across the increased resistance in the *cis* nanopore. We hypothesize that this reduces the effective capture volume, thereby decreasing the likelihood of capturing a second nucleosome near the *trans* nanopore. To test this, we increased the loading bias to +200 and +300 mV. The stronger electric field facilitated the sequential loading of multiple nucleosomes within a short time, as evidenced by stepwise drops in ionic current and corresponding fluctuations in fluorescence intensity (Figure S6). We attribute these optical signal fluctuations to the rapid accumulation of multiple fluorophores, which exceeded the microscope’s resolution.

### Controlled Trapping and Release of Multiple Nucleosomes

To achieve controlled loading of multiple nucleosomes, we incrementally increased the loading bias voltage (Figure 2a). Under an initial +100 mV bias, an open-pore baseline ionic current of 5.4 nA was observed (Figure 2b). Trapping of the first nucleosome reduced the ionic current to 4.8 nA, accompanied by a marked increase in fluorescence intensity from baseline to level (i), correlating with the current decrease. Increasing the loading bias to +200 mV elevated the ionic current to 11.6 nA, consistent with the baseline level for a single trapped nucleosome. Subsequent capture of a second nucleosome decreased the current to 10.7 nA, while the fluorescence intensity rose from level (i) to level (ii), confirming the presence of two single-labeled nucleosomes within the nanocavity. Upon removal of the loading bias, the two nucleosomes remained stably trapped for ∼10 s before one escaped, indicated by a decrease in fluorescence intensity to level (iii), matching the intensity initially observed for a single trapped nucleosome (level (ii)). Release of the remaining nucleosome was achieved by applying a reverse bias of −100 mV, as evidenced by an increase in ionic current and a further reduction in fluorescence intensity. However, escape dynamics in multimolecule trapping systems are complex and may depend on factors such as molecular arrangement, intermolecular interactions, and local surface effects of the nanopore gate. A more systematic study of these behaviors will be an important direction for future work.

**Figure 2:**
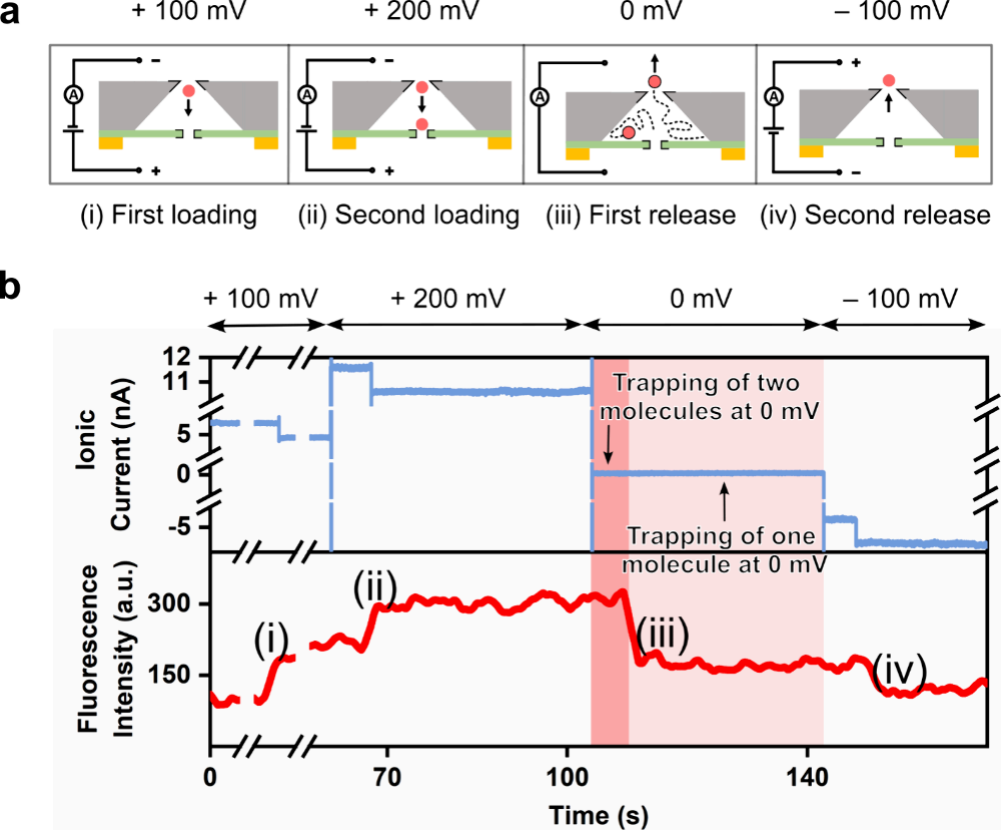
Controlled trapping and release of multiple nucleosomes using a stepwise voltage ramp. (a) Schematic representation of the sequential loading and release process. (i) The first nucleosome is trapped under a +100 mV bias. (ii) Increasing the voltage to +200 mV enables the capture of a second nucleosome. (iii) Removal of the voltage creates confinement in the absence of the external force, during which one nucleosome escapes while the other remains trapped. (iv) Applying a reverse bias of −100 mV releases the remaining nucleosome. (b) Time traces of ionic current (blue) and Cy5 fluorescence intensity (red) corresponding to the steps described in (a). Data were recorded for 1 nM fluorophore-labeled nucleosomes in imaging buffer. Dark pink shading indicates trapping of two nucleosomes, while light pink shading represents trapping of a single nucleosome.

### The Nanocavity Enables Single-Molecule FRET Measurements

Next, we sought to demonstrate the capability of our device to enable single-molecule FRET measurements on nucleosomes confined within the nanocavities. To enhance data acquisition efficiency, we employed a 5 × 5 nanocavity array (Figure S7). However, the optical signal from each nanocavity was still analyzed independently, treating each as a single nanocavity device. In contrast, the ionic-current signal measure from the nanocavity array was less sensitive than that obtained from the single nanocavity. To validate that our nanocavity enables FRET-based measurements of relative distances on the nanometer scale, we reconstituted two types of nucleosomes. Each contained a 78-bp DNA linker extending from one side of the histone core, with a Cy3 donor fluorophore conjugated to histone H2A­(K120C) and a Cy5 acceptor fluorophore positioned at the 5′ end of the short DNA linker on the opposite side. The nucleosome constructs differed in that they contained either a 3-bp or a 19-bp DNA linker, which altered the proximity of the donor and acceptor fluorophores (Figure 3a). Single-step photobleaching events observed with nanocavity-loaded nucleosomes confirmed the presence of single Cy3 donor and Cy5 acceptor dyes (Figure S8). To eliminate the influence of the electric field, we measured Cy3 and Cy5 fluorescence emissions from individual nucleosomes trapped within the nanocavity after removing the voltage, with Cy3 donor excitation performed using a 532 nm laser (Figure 3b). The mean FRET values for nucleosomes with 3-bp or 19-bp short DNA linkers differed substantially (0.393 and 0.218, respectively) (Figure 3b), consistent with the closer proximity of donor and acceptor fluorophores in the 3-bp linker construct.

**Figure 3:**
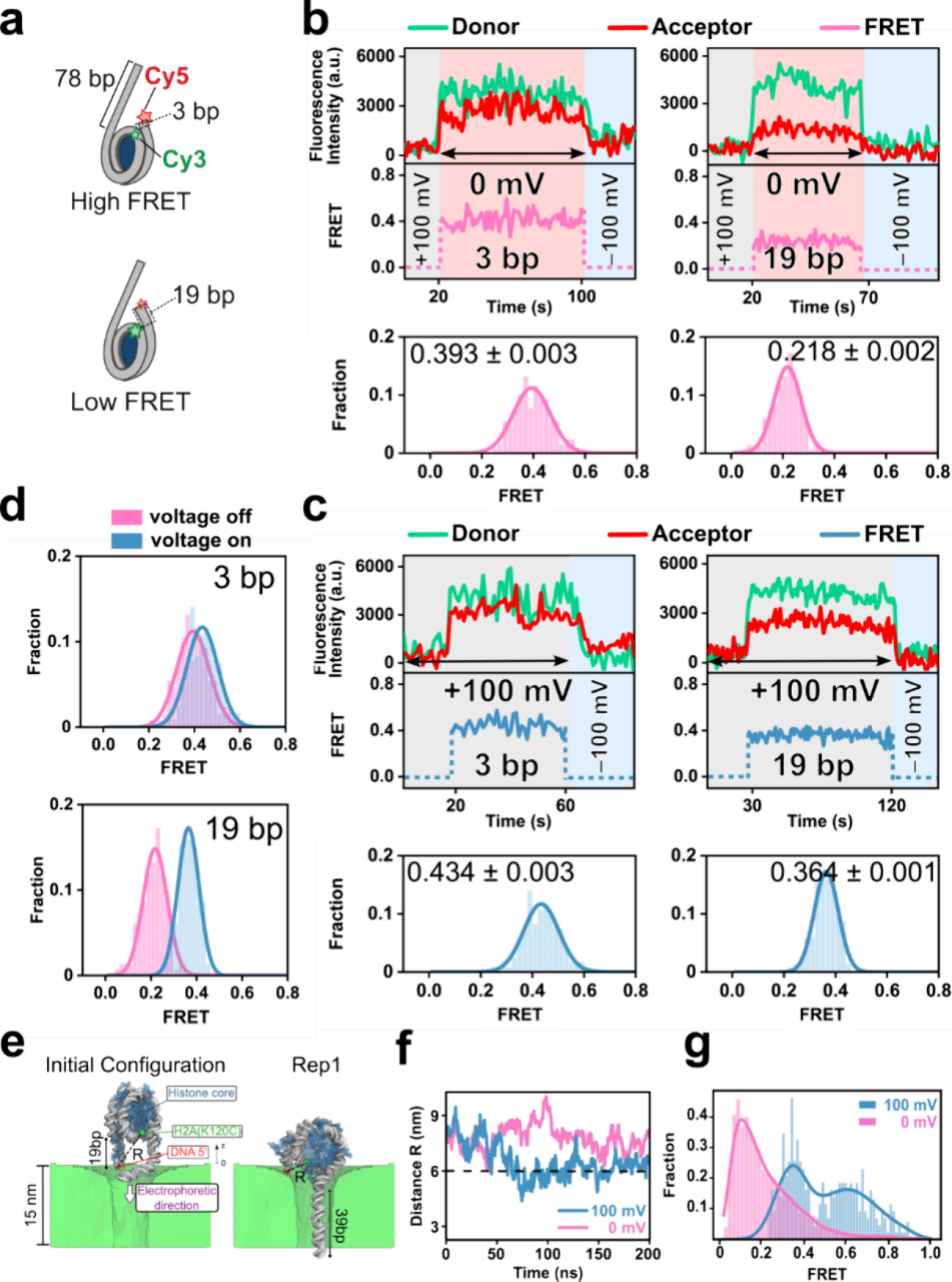
Effect of the electric field on nucleosome conformation. (a) Schematic representations of nucleosomes containing either a 3-bp or a 19-bp DNA linker. (b) Representative time traces of donor (green), acceptor (red), and FRET (pink) signals for 3-bp and 19-bp nucleosomes recorded at 0 mV. Histograms of the mean FRET distributions for 3-bp (*N* = 40 events from 5 independent experiments) and 19-bp (*N* = 45 events from 5 independent experiments) nucleosomes in the absence of voltage. Shading represents conditions: gray for +100 mV, pink for 0 mV, and blue for −100 mV. (c) Representative time traces of donor (green), acceptor (red), and FRET (blue) signals for 3-bp and 19-bp nucleosomes recorded at +100 mV. Histograms of the mean FRET distributions for 3-bp (*N* = 43 events from 5 independent experiments) and 19-bp (*N* = 38 events from 5 independent experiments) nucleosomes under voltage. (d) Gaussian-fitted histograms of mean FRET values for 3-bp and 19-bp nucleosomes, with and without applied voltage. (e) Molecular dynamics simulation of a nucleosome with 19-bp linker captured at the *cis* nanopore of the setup shown in Figure 1. The Rep1 panel displays the final frame of one independent replicate under an applied electric potential +100 mV; the other two replicates, under the same conditions, are shown in Figure S10. (f) Time evolution of the distance (R) between the fluorophore attachment sites for replicate 1. The black dashed line marks the Förster distance (*R*_0_), indicating the distance at which energy transfer is 50% efficient. Note that the fluorophores themselves are not explicitly modeled in the MD simulations, thus, the measured distance reflects only the attachment site locations. Furthermore, *R*_0_ depends on the relative orientation of the fluorophores. (g) FRET values derived from the distances computed in three MD replicates after 150 ns, assuming a fixed *R*_0_.

### Influence of Electric Field on Nucleosome Conformation

Electric fields could perturb biomolecular conformation, destabilizing metastable states and reducing their lifetimes under high field strength.^67,68^ FRET provides a sensitive tool to detect such conformational changes on the nanometer scale. Since our nanocavity allows the observation of single nucleosomes both with and without an applied electric field, we reason that combining the nanocavity device with smFRET analysis would enable the investigation of the electric field’s impact on the conformation of trapped nucleosomes. For direct comparison, we therefore also constructed mean histograms for both nucleosome types during trapping under a maintained +100 mV bias (Figure 3c). The mean FRET value for the 3-bp linker nucleosome remained constant at approximately 0.39, regardless of the presence or absence of the electric field (Figure 3d). In contrast, the mean FRET value for the 19-bp linker nucleosome increased substantially, from ∼0.21 in the absence of an electric field to 0.36 under +100 mV bias. This marked increase in FRET efficiency indicates that the 19-bp DNA linker bends toward the histone core under the influence of the electric field, bringing the fluorophores into closer proximity. By comparison, the 3-bp linker is too short to undergo similar deformation. The effect of the applied voltage on nucleosome conformation is challenging to predict using simple assumptions, given the highly charged nature and asymmetry of the tested complexes. These results were also consistent across different devices as displayed in Figure S9.

To better understand how the applied electric field modulates the fluorophore distance in both constructs, we performed a series of all-atom molecular dynamics (MD) simulations for the 19-bp and 3-bp linker cases. The configuration of the system is shown in Figure 3e. The nucleosome is modeled near the entrance of the *cis* nanopore (green) in the setup illustrated in Figure 1a and is simulated under an electric field corresponding to +100 mV or without any applied field. For the 19-bp linker, the simulations revealed that the reduction in fluorophore distance under the applied voltage arises from a combination of electric pulling forces acting on the longer 78-bp (39-bp in the simulation box) DNA tail and the confinement of the nucleosome at the pore (blue line in Figures 3f and S10c,d). Specifically, the electric pulling force exerted on the longer DNA tail through the pore drives the nucleosome against the pore walls. This interaction, combined with the funnel-shaped entrance, causes the 19-bp DNA linker to bend around the histone core. In all the three MD replicas we performed, the fluorophore distance decreased as the nucleosome became trapped at the nanopore. After 150 ns, the distance stabilized, oscillating around the Förster distance (6 nm), as shown in Figure 3f (MD mean 6.2 ± 0.4 nm). Notably, the distance distribution exhibited a peak at around 6.5 nm, corresponding to an average FRET efficiency of 0.38 (Figure 3g). As a control, we also simulated the nucleosome without any applied voltage or confinement, within an open water box (pink line in Figures 3f and S10e). In this case, the fluorophore distance (7.7 ± 0.4 nm) was consistently larger than the Förster distance, with a corresponding calculated FRET efficiency of 0.2 ± 0.1 (Figure 3g). For the 3-bp linker, the shorter DNA segment constrains the interdye geometry from the outset. The fluorophore distance was already close to the Förster radius, even in the absence of applied voltage, with an average distance of 5.7 ± 0.5 nm (Figure S11c) and a corresponding average FRET efficiency of 0.58 ± 0.12 (Figure S11e). Under +100 mV, the electric field and pore confinement produced only a modest further reduction in distance to 5.5 ± 0.4 nm (Figure S11b), corresponding to an average FRET efficiency of 0.63 ± 0.10. This contrasts with the much larger field-induced compaction observed for the 19-bp DNA linker. The smaller shift between the two voltage conditions is consistent with the limited conformational freedom of the shorter 3-bp DNA linker, which leaves little room for additional field-driven bending. In both constructs, the computed FRET efficiencies are in good agreement with the experimental results (Figure 3d).

### Observation the Dynamics of Individual Nucleosomes and Weak Interactions of Two Nucleosomes Inside the Nanocavity

To evaluate the ability of our nanocavity device to monitor the dynamics of individual molecules using single-molecule FRET, we investigated nucleosomal DNA unwrapping and rewrapping dynamics induced by the Chd1 chromatin remodeler.^69^ We reconstituted nucleosomes with a donor fluorophore (Cy3) attached to the long DNA linker and an acceptor fluorophore (Cy5) attached to the short DNA linker, positioning the fluorophores in close proximity (Figure 4a). In this labeling scheme, unwrapping of the outer DNA gyre from the nucleosome increases the distance between the fluorophores, resulting in a decrease in FRET efficiency. Upon trapping nucleosomes in the nanocavity, we recorded fluorescence signals from individual complexes in the presence of Chd1 and the nonhydrolyzable ATP analog ATPγS, with no applied electric field (Figure 4b). FRET time traces revealed repeated transitions between high- and low-FRET states, indicative of a dynamic equilibrium between fully wrapped and unwrapped DNA conformations. In contrast, fluorescence signals from individual nucleosomes in the absence of Chd1 (Figure S12) did not show any FRET state transitions. These observations align with the known ability of Chd1 to transiently and reversibly unwrap the outer DNA gyre from the nucleosome.^70^ In addition, we reconstituted nucleosomes labeled with either Cy3 or Cy5 (Figure 4c) and demonstrated the sequential loading of a Cy3-labeled and a Cy5-labeled nucleosome from a mixed solution, along with analysis of their weak interactions in the absence of an electric field. As shown in Figure 4d, the donor (green) intensity initially increased, confirming the loading of a Cy3-labeled nucleosome at +100 mV. Increasing the loading voltage to +200 mV subsequently trapped a Cy5-labeled nucleosome. We observed the association between two nucleosomes through the decrease in donor and simultaneous increase in acceptor (red) intensity which may arise from stacking interactions between the two nucleosomes.^71–74^ Upon removal of the electric field, the FRET efficiency (black) gradually decays, showing their slow dissociation (Figure 4c). The same dissociation trend upon removal of the electric field was also observed for a prestacked nucleosome complex loaded into the nanocavity (Figure S13a). In contrast, the FRET efficiency increased in the presence of an electric field (Figure S13b). Thus, our nanocavity device enables trapping of individual macromolecular complexes without the application of an external force and facilitates real-time observation of their molecular dynamics and weak interactions.

**Figure 4:**
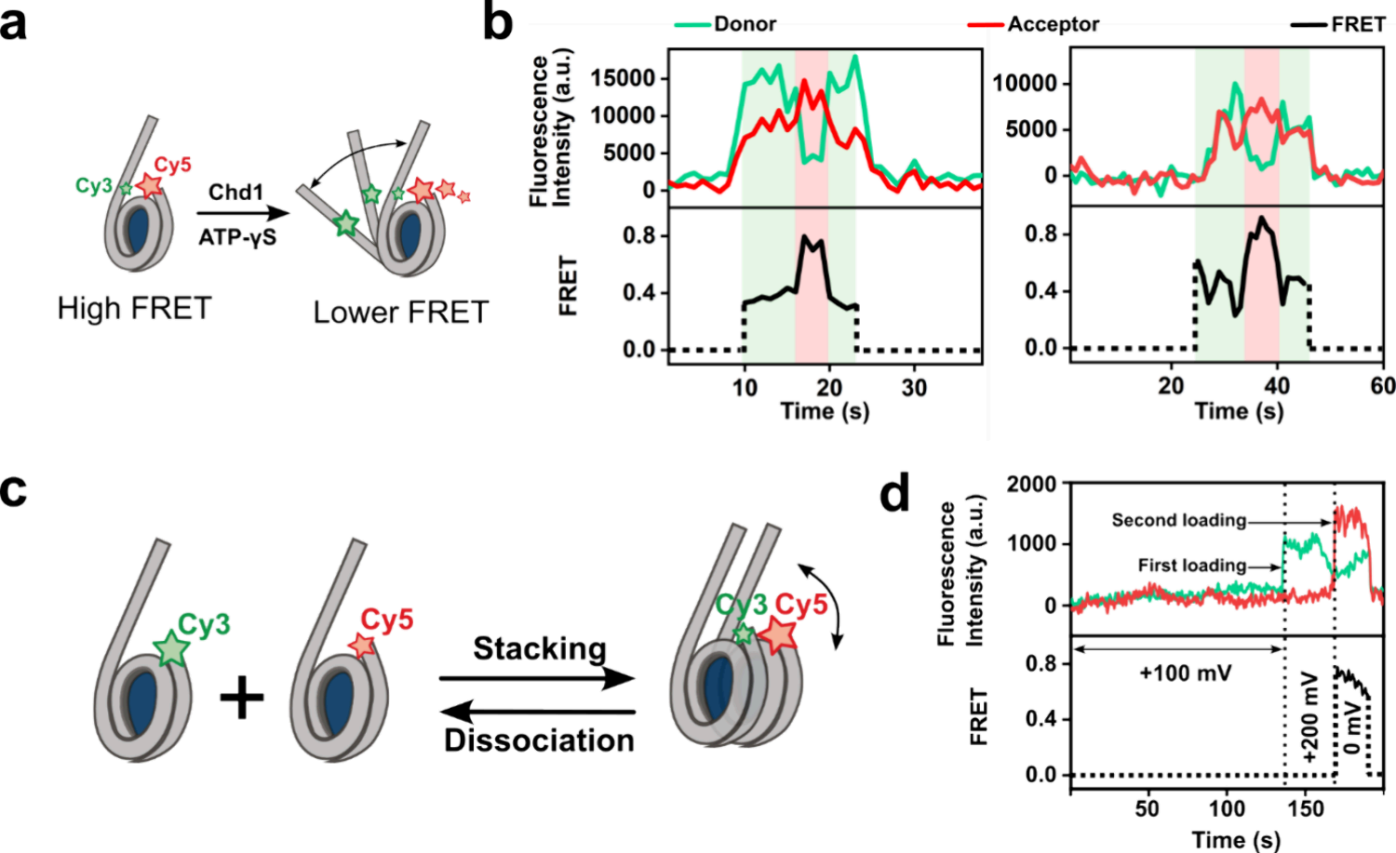
Nucleosomal DNA unwrapping by the chromatin remodeler Chd1 and weak interaction between two nucleosomes. (a) Schematic of the labeling scheme and the dynamic equilibrium between fully wrapped and unwrapped nucleosome conformations in the presence of Chd1 and ATPγS. (b) Representative time traces of donor fluorescence (green), acceptor fluorescence (red), and FRET efficiency (black) illustrating Chd1-induced unwrapping and rewrapping of individual nucleosomes in the presence of ATPγS, recorded while the nucleosomes were confined in the nanocavity at 0 mV. Shading indicates states: green for lower-FRET and red for higher-FRET states. (c) Schematic of the weak interaction between two nucleosomes labeled with donor and acceptor, respectively. The conceptual cartoon is not meant to imply any particular mode of nucleosome stacking. The double-headed black arrow illustrates the dynamic nature of stacked nucleosomes: while they can form stable interactions, they undergo relative motions (*e.g.*, tilting and rotational fluctuations). (d) Representative time traces of donor fluorescence (green), acceptor fluorescence (red), and FRET efficiency (black) illustrating the dynamics of two nucleosomes sequentially loaded from a mixed solution.

## Conclusions

In this work, we present a nanopore-gated, subattoliter silicon nanocavity device with customizable pore sizes, designed for single-molecule trapping and analysis without applying an external force. To accommodate the 11 nm size of the nucleosome, our model molecule, we developed an electron beam-induced carbon deposition technique to precisely reduce the nanopore gate sizes to 12 and 9 nm. Using ionic current as real-time feedback, we achieved controlled loading of single or multiple nucleosomes into the nanocavity. The nucleosomes were stably trapped inside the nanocavity through entropy-driven confinement, requiring no applied external forces which represents the main improvement over conventional nanopore studies. Our single-molecule FRET analyses of donor–acceptor-labeled nucleosomes showcase the capability of the device to probe the dynamics of biologically important macromolecular complexes under defined conditions. Our data further demonstrate that an applied electric field can modulate the conformational properties of the macromolecules, emphasizing a key advantage of our device: it does not require an electric field to retain trapped molecules. Furthermore, the nanocavity devices were fabricated using standard silicon technology, enabling scalability and cost-effective mass production in conventional chip manufacturing foundries. By confining individual molecules within a subattoliter volume, the nanocavity achieves effective concentrations approaching 10 μM for a single molecule. We anticipate this capability to facilitate the study of biological processes requiring high local concentrations, while also addressing traditional challenges such as signal saturation in fluorescence-based approaches. Meanwhile, we notice the practical limitations of this approach at current stage, including the requirement that the target molecule be large enough to be retained within the selected gate geometry. Functionalizing the top nanopore with polymer brushes^75^ to enhance the entropic barrier could be a future research direction to address these limitations. Consequently, the nanocavity will enable the investigation of weak molecular interactions at the single-molecule level that may otherwise evade detection. We therefore envision this nanocavity platform as a powerful tool for the interrogation of molecular dynamics without applying an external force, offering unperturbed access to weak and transient interactions central to biological regulation.

## Methods

### Nanopore-Gated Nanocavity Fabrication

The fabrication process of the nanopore-gated nanocavity device began with a double-side-polished (100) silicon-on-insulator (SOI) wafer. The SOI wafer consisted of an 88 nm-thick top silicon (Si) layer, a buried 145 nm-thick oxide (BOX) layer, and a 300-μm-thick Si substrate. First, a 30 nm-thick low-stress silicon nitride (SiN_*x*_) layer was deposited on both sides of the SOI wafer using low-pressure chemical vapor deposition (Koyo Lindberg). Photolithography (Karl Süss) and reactive ion etching (RIE; Advanced Vacuum) were used to open square windows (150 μm per side) on the SiN_*x*_ layer from the bottom side of the wafer. Subsequently, large cavities in the Si substrate were etched using a combination of deep RIE and wet etching in 80 °C KOH. During this step, the top SiN_*x*_ layer was protected, and the BOX layer served as an effective etch stop for the KOH etching. Next, a 50 nm-thick gold layer was deposited onto the top SiN_*x*_ layer using an evaporator (Kurt J. Lesker Company) and lifted off, leaving an uncovered SiN_*x*_ region aligned with the backside window. Nanoscale holes were patterned in the uncovered top SiN_*x*_ layer via electron beam lithography (Nanobeam Ltd.) followed by RIE. Nanocavities were then formed by etching the top Si layer in 60 °C KOH through the nanoscaled holes and stripping the BOX layer. The lateral etching of the (111) Si planes primarily controlled the formation of the nanocavities. Finally, the fabricated chips were mounted on adhesive carbon tabs and loaded into a scanning electron microscope (SEM; Zeiss 1530, Germany) chamber for electron beam-induced carbon deposition.^76^ More than 20 chips with similar device dimensions were fabricated, the precise size control was confirmed by high-resolution TEM analysis of three representative devices.

### Preparation of Fluorophore-Labeled Nucleosomes and TOTO-1 Labeled DNA

Recombinant histones (H2A, H2A­(K120C), H2B, H3 C110A and H4) from*Xenopus laevis* were purchased from the Histone Source Protein Expression and Purification Facility, Colorado State University, Fort Collins, CO. H2A­(K120C) was labeled with Cy3 as described previously.^77^ Briefly, one milligram of lyophilized H2A120C was diluted in unfolding buffer (20 mM Tris pH 7.0, 7 M guanidine-HCl, 5 mM EDTA, 1.25 mM TCEP) and incubated for 2 h at room temperature in the dark. Cy3-maleimide was dissolved in DMSO and added to the protein at a final concentration of 0.75 mM. After 3 h in the dark at room temperature, the reaction was quenched with a final concentration of 80 mM β-mercaptoethanol. The labeled protein was dialyzed nine times against dialysis buffer (20 mM Tris pH 7.0, 7 M guanidine-HCl, 1 mM DTT) and then used in histone dimer assembly. The labeling efficiency was approximately 70–85%.

Biotinylated and fluorescently labeled DNA constructs containing the Widom 601 nucleosome positioning sequence were generated by PCR (for +3 and +19 nucleosomes) or by annealing and ligating a set of overlapping oligonucleotides (unwrapping nucleosomes).^78^ These DNA constructs have a long (78-bp) stretch of flanking DNA with biotin at the end on one side of the Widom 601 nucleosome positioning sequence and a short stretch of either 3 or 19 bp of flanking DNA with a Cy5 fluorophore at the end on the other side of the nucleosome positioning sequence. The unwrapping construct additionally has an internal Cy3 DNA label on the long-linker side located 4 bp away from the edge of the nucleosome positioning sequence. “Cy3-only” and “Cy5-only” DNA constructs used to monitor nucleosome dimerization have a 63-bp linker on one side and no linker on the other side of the Widom 601 nucleosome positioning sequence, with either a Cy3 or a Cy5 fluorophore attached to the 5′ DNA terminus on the “no-linker” side, and were generated by PCR.

Unwrapping, “Cy3-only” and “Cy5-only” nucleosomes were directly reconstituted by salt gradient dialysis using unlabeled histone octamer and purified by preparative polyacrylamide gel electrophoresis using the Mini Prep Cell apparatus (Bio-Rad), as described previously.^78^ Briefly, 2 μM nucleosomal DNA was mixed with 2.4 μM histone octamer in the assembly buffer (20 mM Tris-HCl pH 7.5, 1 mM EDTA, 10 mM DTT and 2 M KCl) and incubated for 20 min on ice before loading into a dialysis cassette (7 kDa cutoff, Thermo). Nucleosome assembly is carried out at 6 °C. The cassette was placed into 250 mL of high-salt buffer (10 mM Tris-HCl pH 7.5, 2 M KCl, 1 mM EDTA and 1 mM DTT) which was slowly diluted with 800 mL of low-salt buffer (10 mM Tris-HCl pH 7.5, 250 mM KCl, 1 mM EDTA and 1 mM DTT) while keeping the total volume constant over the course of approximately 20 h. Then the cassette was dialyzed against the low-salt buffer for 2 h and against TCS buffer (20 mM Tris-HCl pH 7.5, 1 mM EDTA and 1 mM DTT) for another 2 h. Nucleosomes were purified on a 7% native polyacrylamide gel column (60:1 acrylamide:bis­(acrylamide) ratio) in 0.5x TBE buffer (44.5 mM Tris pH 7.6, 44.5 mM boric acid, 1 mM EDTA) on a Mini Prep Cell apparatus (Bio-Rad). Purified nucleosomes in elution buffer (15 mM HEPES pH 7.5, 1 mM EDTA, 0.5 mM TCEP) were concentrated to 1–2 μM using Microcon 50K spin-concentrators, supplemented with 5% glycerol, flash-frozen in liquid nitrogen and stored at −80 °C for long-term storage. Thawed nucleosome aliquots were stored at 4 °C for up to several months.

“Cy3-Cy5-both” nucleosomes were assembled in the same way as “Cy3-only” and “Cy5-only” nucleosomes using the “Cy5-only” DNA and histone octamer containing H2A120C-Cy3 instead of the wild-type H2A. + 3 and +19 nucleosomes^79^ were reconstituted by adding wild-type H2A/H2B dimer to oriented hexasomes assembled with H2A120C-Cy3 as described previously.^80^ Briefly, the assembly and polyacrylamide purification process was the same as for “Cy3-only” and “Cy5-only” nucleosomes, but instead of histone octamer, H3/H4 tetramer and H2A120C-Cy3/H2B dimer were added to nucleosomal DNA separately, with 1.2:1 tetramer-to-DNA ratio and 1.2:1 dimer-to-tetramer ratio (as opposed to 2:1 dimer-to-tetramer ratio in a histone octamer) in order to favor hexasome formation. To reconstitute complete +3 and +19 nucleosomes, wild-type H2A/H2B dimer was added to purified +3 and +19 hexasomes at 1.2:1 ratio and incubated at room temperature for at least 5 min before reconstituted nucleosomes were used in experiments. Reconstituted nucleosomes were stored at 4 °C for up to several months.

Twenty kb DNA ladders were purchased from Fisher Scientific. TOTO-1, an intercalating fluorescent DNA dye, was used to label the DNA molecules with a nucleotide to dye ratio of 10:1.

The imaging buffer contained 20 mM Tris pH 7.0, 100 mM KCl, 0.1 mg/mL acetylated BSA (Promega), 10% (v/v) glycerol, 10% (w/v) glucose, supplemented with 2 mM Trolox to reduce photoblinking of the dyes, as well as an enzymatic oxygen scavenging system (composed of 800 μg/mL glucose oxidase and 50 μg/mL catalase). Nucleosomes were then dispersed in the imaging buffer to 1 nM and DNA to 100 pM, respectively. To study the dynamics of individual nucleosomes, 0.4 μM Chd1, 1 mM MgCl_2_ and 1 mM ATP γS were added together with unwrapping nucleosomes. To monitor the dynamics of nucleosome dimerization, Cy3-only nucleosomes, Cy5-only nucleosomes and 1 mM MgCl_2_ were mixed together. Both nucleosomes and DNA were negatively charged in all experiments.

### Electrical and Optical Characterizations

Prior to the electrical measurements, the nanopore chip was carefully cleaned in oxygen plasma at 1,000 W for 10 min, followed by immersion in a piranha solution (H_2_SO_4_:H_2_O_2_ = 3:1 in volume) for 30 min and finally rinsed in deionized water. The nanopore chip was then mounted on a custom-made poly­(methyl methacrylate) flow cell (Figure S14) and the *cis* chamber was sealed using a 0.17 mm thick cover glass. Imaging buffer containing 0.1 mg/mL BSA was then added to both chambers, and the chip was immersed to passivate the solid surface. Two compartments were separated by the chip and the only path of ionic current was through the nanopore. The flow cell was placed on a widefield fluorescence microscope (Ti Eclipse, Nikon) equipped with a 63x water-immersion objective (Nikon). A pair of Ag/AgCl electrodes (2 mm in diameter (Warner Instruments LLC.)) was used to apply a bias voltage across the nanopore and to measure the ionic current. The electrical measurement was monitored using a patch clamp amplifier (Multiclamp 700B, Molecular Device Inc.). The voltage control and current record were achieved with a custom LABVIEW program. The ionic current was digitalized by an Axon Digidata 1550B1 (Molecular Device LLC.) and recorded using the software Axon pCLAMP 11 (Molecular Device LLC.). The traces were sampled at 10 kHz and low-pass filtered at 5 kHz. Extraction of translocation events was performed with the Transalyzer package.^81^ Prior to introducing the actual biological sample, we conducted pre-experiments by sweeping the I–V curve multiple times to confirm the reproducibility of regular ionic signals under episodic voltage applied across the nanocavity. Then the *trans* chamber was replaced with imaging buffer containing target molecules while the *cis* chamber only filled with imaging buffer. TOTO-1 and Cy3 fluorophores were excited with a PE-300 white broad spectrum LED illuminator (CoolLED) through a Cy3 excitation filter. Fluorescence emission in the Cy3 (or TOTO-1) and Cy5 spectral channels is projected side-by-side onto an EMCCD camera (iXon 888 Ultra, Andor). Images were processed and analyzed by using Fiji/ImageJ software.^82,83^ The electrical time resolution in the present experiments was limited by the 5 kHz filter frequency. However, the main purpose of the nanocavity platform is to enable prolonged observation of trapped molecules in the absence of an applied external force, rather than continuous high-bandwidth electrical detection. For optical measurements, the time resolution is primarily determined by the fluorescence detection method rather than the nanocavity itself. In this study, camera-based imaging below 10 Hz was used for proof-of-concept experiments, although substantially higher time resolution can be achieved using faster fluorescence detection approaches.

### Molecular Dynamics Simulation

The simulation box is composed of a neutral solid-state membrane (green) containing a drilled nanopore and a reduced version of the nucleosome used in the experimental setup. The nucleosome model includes histones (transparent blue), 147-bp of double-stranded DNA (dsDNA) wrapped around the histone, and two elongated dsDNA tails: a shorter tail of 19-bp or 3-bp and a longer tail of 39-bp. The residues where the two fluorophores are attached, Cy3 (green, located on H2A­(K120C)) and Cy5 (red, at the DNA 5′ end), are highlighted. To reduce computational cost, the modeled membrane thickness and the length of the longer DNA tail are halved in comparison to the experimental values (30 nm and 78-bp, respectively). Initially, the nucleosome is positioned at the nanopore entrance. After equilibration, a constant, homogeneous electric field is applied along the *z*-axis, *E* = (0,0,Ez), simulating an applied voltage of +100 mV, as done in other works.^84,85^ The negatively charged long tail is electrophoretically pulled through the nanopore. After 150 ns, the nucleosome reaches a stable state, with the center of mass of the protein core located between 3 and 5 nm above the membrane surface (Figures S10f and S11d).

The formula used to convert MD distances to FRET values (*R*_0_ = 6 nm) is

$$\mathrm{FRET}=\frac{1}{1+{\left(\frac{R}{R_{0}}\right)}^{6}}$$

## Supplementary Material



## Notes

Process flow for fabricating the nanopore-gated nanocavity device (Figure S1), relationship between nanopore gate size and dwell time of 20 kb DNA in the nanopore-gated nanocavity (Figure S2), pore size reduction (Figure S3), current–voltage (I–V) characteristics (Figure S4), nucleosome trapping (Figure S5), sequential trapping of two fluorophore-labeled nucleosomes (Figure S6), image of nanocavity array (Figure S7), single-step photobleaching measurements (Figure S8), effect of the electric field on nucleosome conformation in different devices (Figure S9), molecular dynamics simulation for single nucleosomes with 19-bp and 3-bp DNA linker (Figure S10–11), fluorescence intensity and FRET distributions of single nucleosomes with or without Chd1 (Figure S12), weak interaction between two nucleosomes (Figure S13), schematic of the custom-fabricated flow cell (Figure S14), supplementary method of MD simulation (PDF)

## ∇

Department of Radiology, Stanford University, Stanford, California 94305 United States

## ○

School of Integrated Circuits, Huazhong University of Science and Technology, Wuhan 430074, China.
