# Supplementary material for: A Nanopore-Gated Subattoliter Silicon Nanocavity for Single-Molecule Trapping and Analysis without Applying an External Force
Source: ACS Nano. 2026 Jun 22;20(26):19096–106. doi: 10.1021/acsnano.6c06884 (PMC13348167; doi:10.1021/acsnano.6c06884)
Supplement: Supplementary file 1 [file nn6c06884_si_001.pdf]

Supporting Information

A nanopore-gated sub-attoliter silicon nanocavity for single molecule trapping and analysis without applying an external force

Funing Liu[1], Qitao Hu[1,7], Anton Sabantsev[2], Giovanni Di Muccio[3,4], Shuangshuang Zeng[1,8], Mauro Chinappi[5], Sebastian Deindl[2,6] and Zhen Zhang[1]**

Contents:

Figure S1-14

Supplementary Method

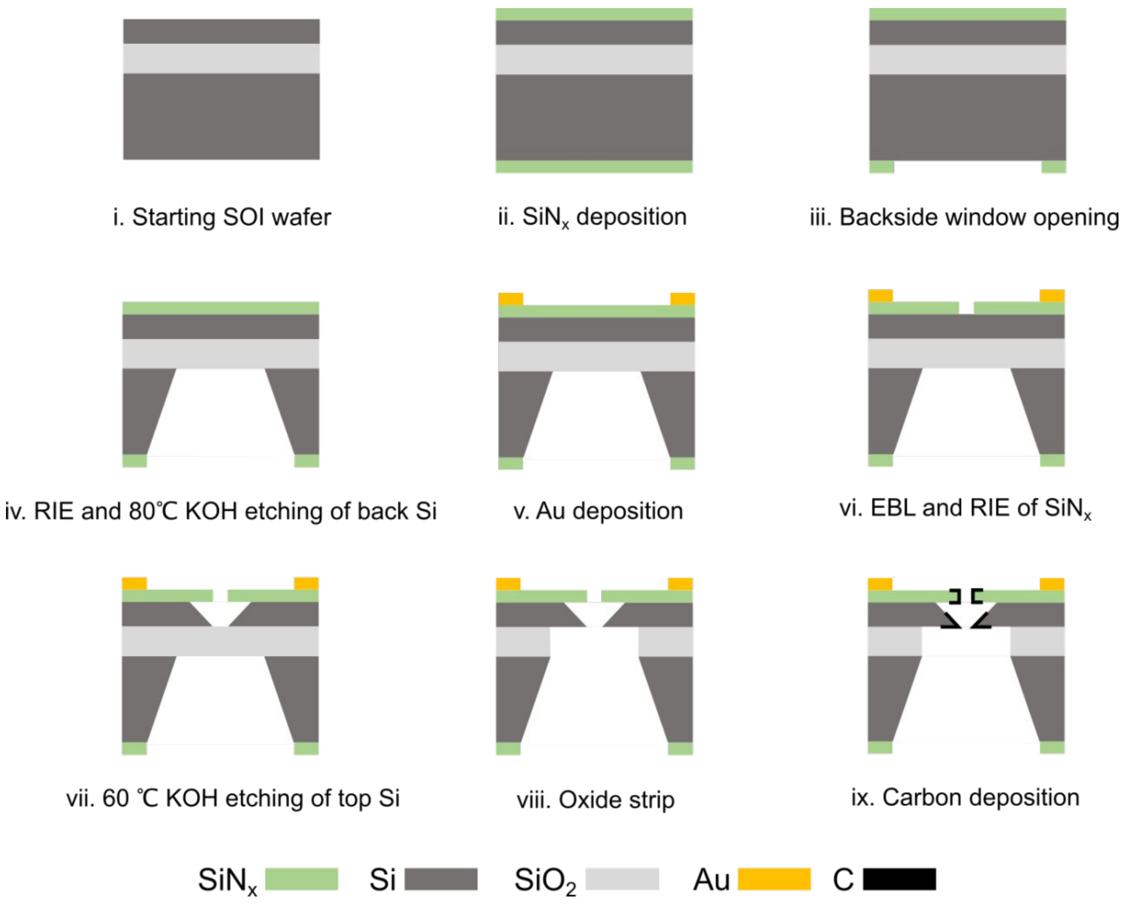

Figure S1. **Schematic illustration of the process flow for fabricating the nanopore-gated nanocavity device.** The fabrication process builds on our established workflow for truncated pyramidal nanopores.[1,2] (i) Starting SOI wafer. (ii) Deposition of SiNx on the SOI wafer using low-pressure chemical vapor deposition (LPCVD). (iii) Backside window opening *via* photolithography, followed by reactive ion etching (RIE). (iv) Silicon etching in the bulk substrate using deep RIE, followed by KOH wet etching at 80 °C. (v) Gold deposition (*via* metal evaporation) and lift-off on the top SiNx layer, leaving an uncovered region aligned with the backside window. (vi) Nanopore creation in the uncovered top SiNx layer using electron beam lithography (EBL) and RIE. (vii) Silicon nanocavity etching in 60 °C KOH solution. (viii) Removal of the buried oxide layer using buffered HF. (ix) Carbon deposition under SEM scanning to precisely tune the nanopore size.

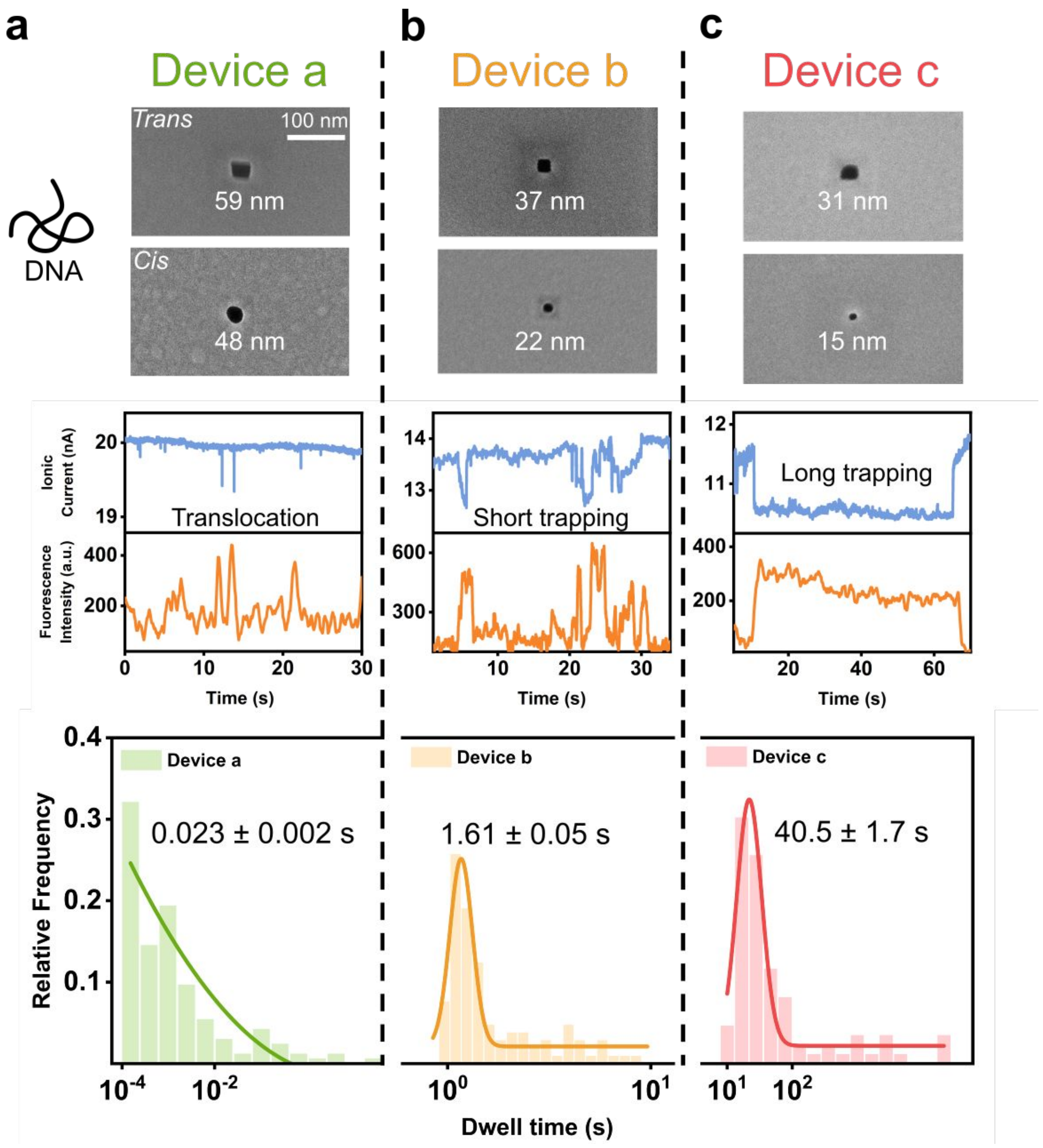

Figure S2. Relationship between nanopore gate size and dwell time of 20 kb DNA during translocation experiments under constant +100 mV bias using the nanopore-gated nanocavities. Three devices with varying *trans* and *cis* nanopore sizes were fabricated to evaluate the dwell times of 20 kb DNA (hydrodynamic diameter approximately 280 nm) under +100 mV bias. Negatively charged DNA molecules in imaging buffer were captured into the nanocavity *via* electrophoretic forces. Top row: SEM images of the devices with *trans* nanopore sizes of 59 nm, 37 nm, and 31 nm and *cis* nanopore sizes of 48 nm, 22 nm, and 15 nm, respectively. Middle row: Representative ionic current (blue) and fluorescence intensity (orange) time traces for 100 pM TOTO-1-labeled 20 kb DNA at +100 mV, showing (a) rapid translocation, (b) short-term trapping, and (c) extended trapping. Bottom row: Dwell time histograms for (a) Device a (green, $N = 70$ events from 5 independent experiments), (b) Device b (yellow, $N = 56$ events from 5 independent experiments), and (c) Device c (red, $N = 32$ events from 5 independent experiments). The histograms were fitted with Gaussian distribution to enable direct comparison across different experimental conditions.

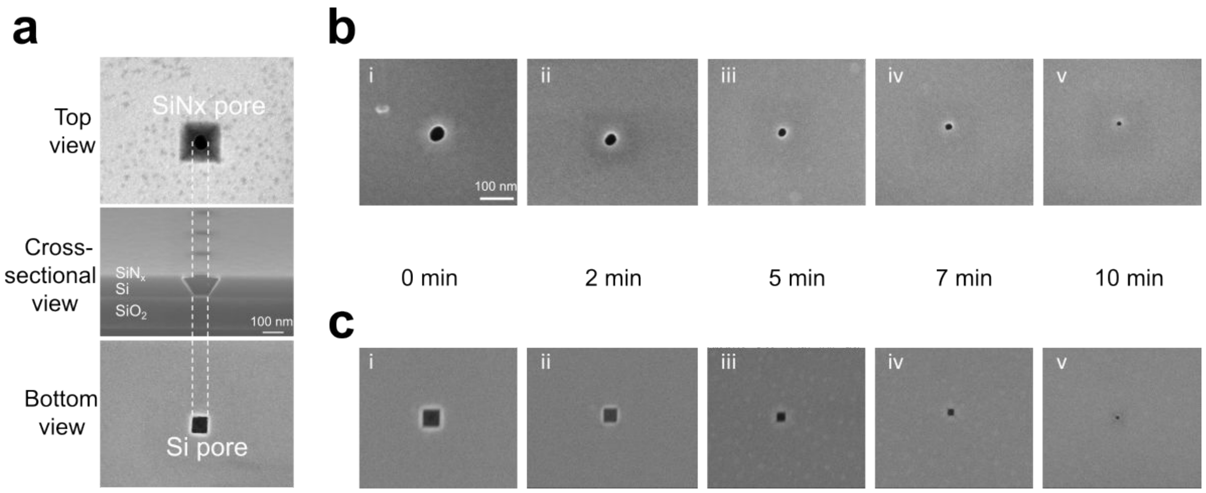

Figure S3. Pore size reduction with real-time monitoring by SEM. During SEM imaging, the hydrocarbon was evaporated by an electron beam from a conductive carbon tab underneath the sample, allowing carbon deposition onto the nanopore surface to reduce the pore size (a) SEM images of a nanocavity fabricated in an 88 nm thick Si membrane, showing top, cross-sectional, and bottom views. (b) Continuous reduction of a 43 nm SiNx pore under SEM at an accelerating voltage of 15 kV and magnification of 400 K: (i) 0 min, (ii) 2 min, (iii) 5 min, (iv) 7 min and (v) 10 min. (c) Continuous reduction of a 46 nm Si pore under the same conditions.

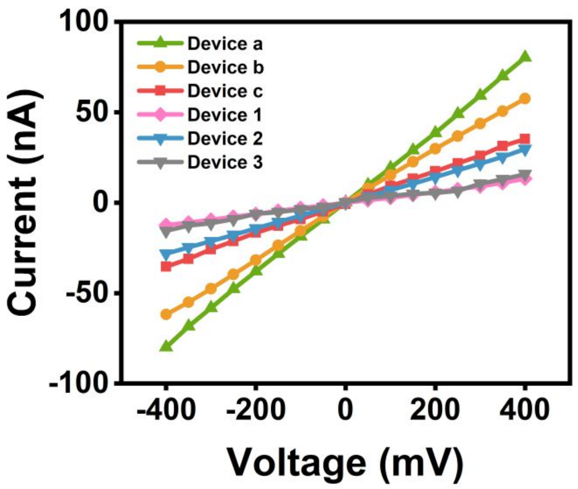

Figure S4. Current-voltage (I-V) characteristics of the six devices in imaging buffer.

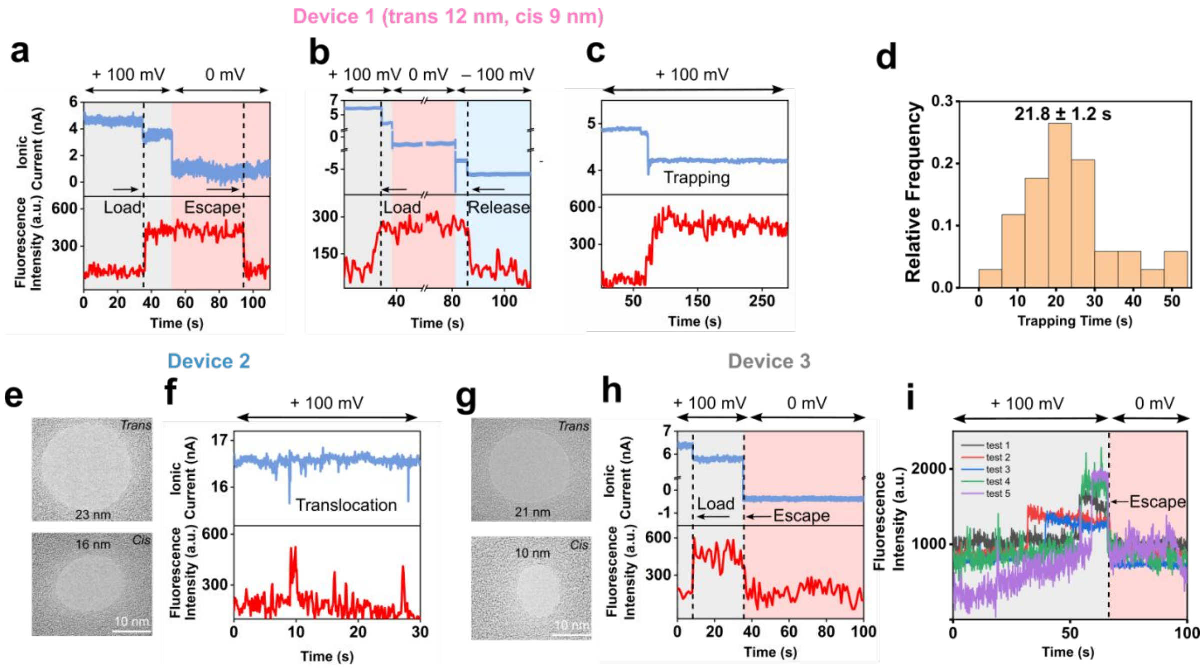

Figure S5. Devices for trapping nucleosomes. Ionic current and fluorescence intensity time traces showing detection of 1 nM fluorophore-labeled nucleosomes in imaging buffer. Data recorded by Device 1 (for TEM images, see Figure 1b): (a) Single nucleosome loading into the nanocavity at +100 mV (grey shading), trapping at 0 mV (pink shading), and subsequent spontaneous escape. (b) single nucleosome loading at +100 mV (grey shading), trapping at 0 mV (pink shading), and subsequent release at –100 mV (blue shading). (c) single nucleosome trapping at a constant +100 mV bias. (d) Trapping time histogram for single nucleosomes at 0 mV (N =72). Data recorded by Device 2: (e) TEM images showing 23 nm *trans* and 16 nm *cis* nanopores. (f) Nucleosome translocation events at a constant +100 mV bias. Data recorded by Device 3: (g) TEM images showing 21 nm *trans* and 10 nm *cis* nanopores. (h) Single nucleosome loading at +100 mV (grey shading) with immediate escape after removal of voltage (pink shading). (i) Independent replicate experiments showing an instantaneous fluorescence intensity decrease upon removal of voltage.

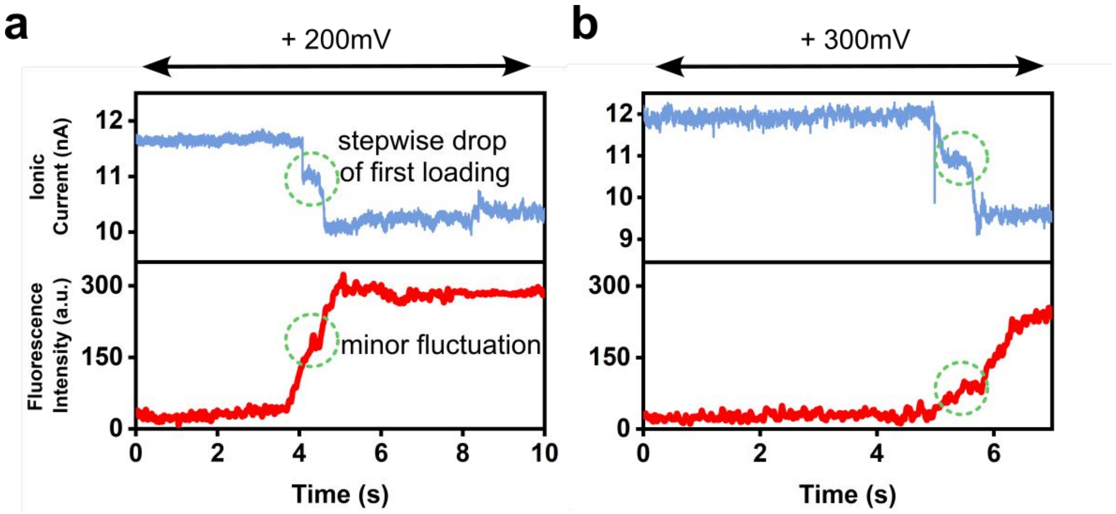

Figure S6. Ionic current and fluorescence intensity time traces showing the sequential trapping of two fluorophore-labeled nucleosomes. The applied voltage was held constantly at (a) +200 mV and (b) +300 mV.

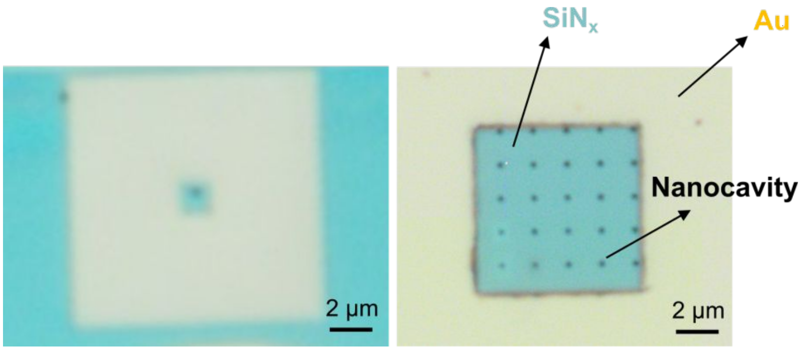

Figure S7. 5 × 5 nanocavity array. Optical micrographs from the *cis* side: single nanocavity (left) and 5x5 nanocavity array (right). Both devices are coated with a gold film.

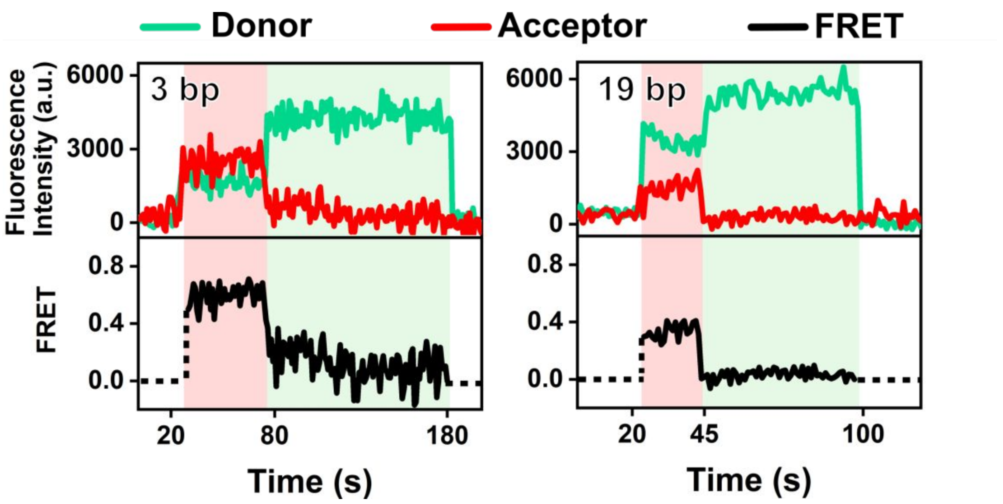

Figure S8. Single-step photobleaching measurements. Representative donor (green), acceptor (red) and FRET (black) time traces recorded in the absence of the oxygen-scavenging system of 3-bp and 19-bp linker nucleosomes. The applied voltage was held constantly at +100 mV. Shaded areas: green indicates lower-FRET and red indicates higher-FRET states.

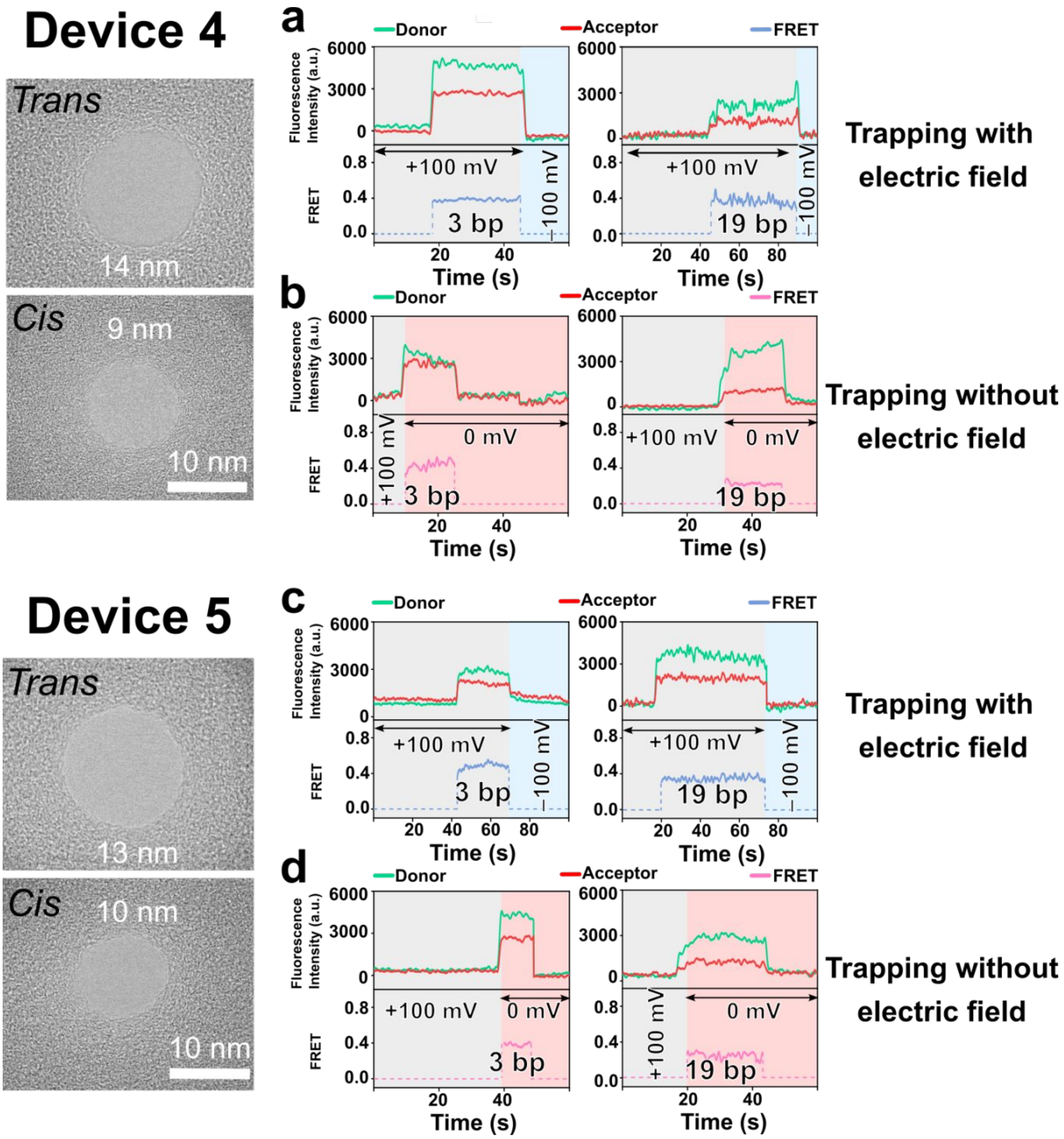

Figure S9. Effect of the electric field on nucleosome conformation in other devices. The left panel displays TEM images of Device 4 (14 nm *trans* and 9 nm *cis* nanopores) and Device 5 (13 nm *trans* and 10 nm *cis* nanopores). Representative time traces of donor (green), acceptor (red), and FRET (blue) signals for 3-bp and 19-bp nucleosomes recorded at +100 mV by (a) Device 4 and (c) Device 5. Representative time traces of donor (green), acceptor (red), and FRET (pink) signals for 3-bp and 19-bp nucleosomes recorded at 0 mV by (b) Device 4 and (d) Device 5. Shaded area color codes: grey for +100 mV, pink for 0 mV, and blue for −100 mV.

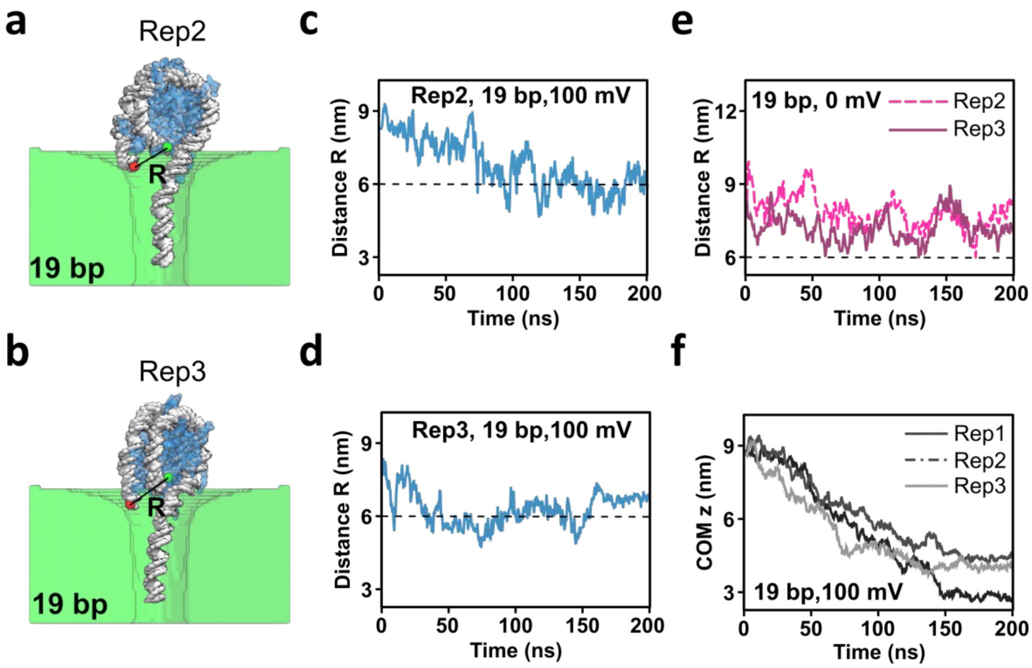

Figure S10. Molecular dynamics simulation for the 19-bp DNA linker. (a-b) Final frame of two independent MD simulations (Rep2 and Rep3) that are replicas of the system reported in Figure 3e of the main manuscript. The MD simulations were performed under an applied electric field E = (0, 0, Ez) corresponding to an electric potential difference of +100 mV across the nanopore. (c-d) Time evolution of the distance R between the fluorophore attachment sites for Rep2 and Rep3, under a simulated voltage of +100 mV. The black dashed line marks the Förster distance (R_0), indicating the distance at which energy transfer is 50%. (e) The same distance R for other two independent simulations without applied voltage or confinement. (f) The center-of-mass (COM) z position of the histone core relative to the nanopore opening over time, for the systems under the applied electric field.

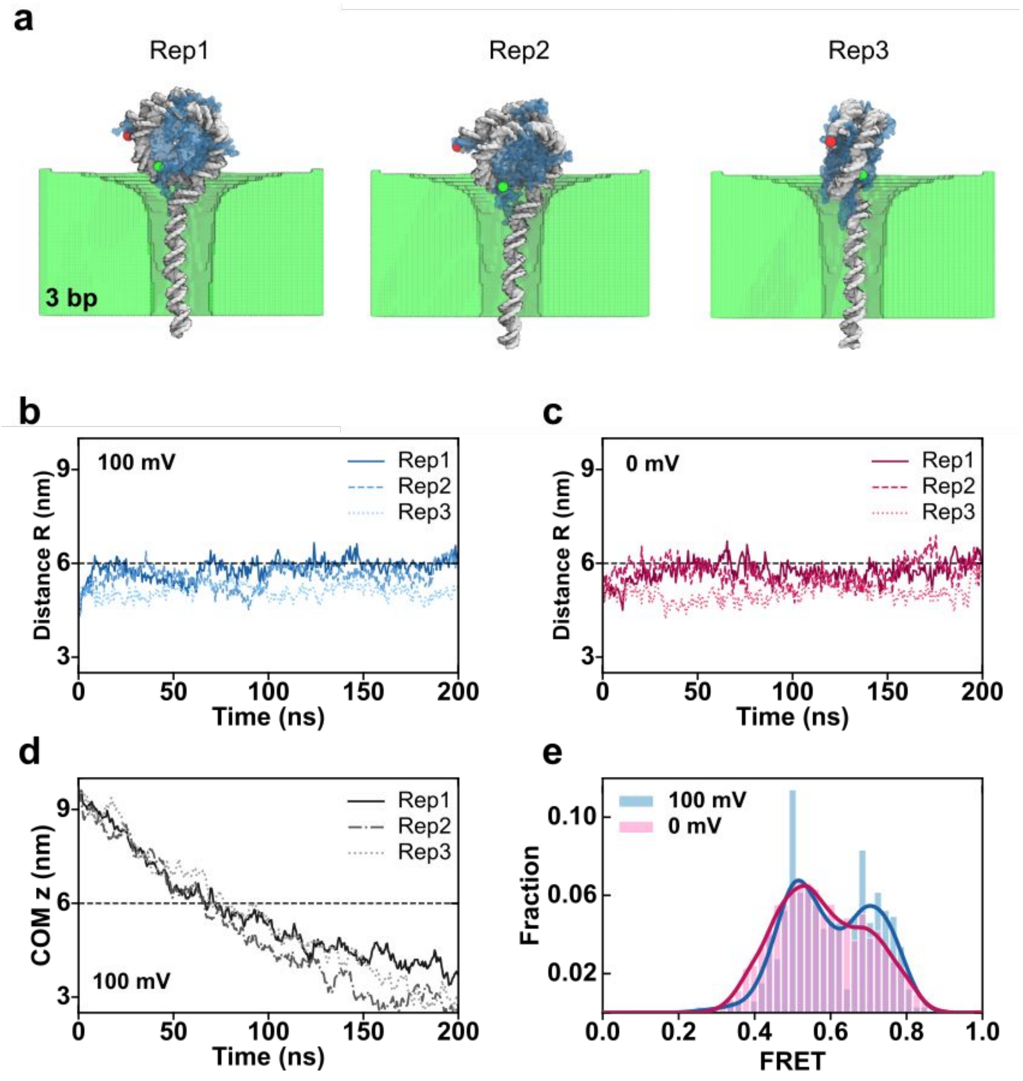

Figure S11. Molecular dynamics simulation for the 3-bp DNA linker. (a) Final frame of three independent MD simulations. The MD simulations were performed under an applied electric field E = (0, 0, Ez) corresponding to an electric potential difference of +100 mV across the nanopore. (b) Time evolution of the distance R between the fluorophore attachment sites under a simulated voltage of +100 mV. The black dashed line marks the Förster distance (R_0 = 6 nm), indicating the distance at which energy transfer is 50%. (c) The same distance R for other three independent simulations without applied voltage or confinement. (d) The center-of-mass (COM) z position of the histone core relative to the nanopore opening over time, for the systems under the applied electric field +100 mV. (e) FRET values derived from molecular distances computed from MD simulations, for the 3 bp constructs, with and without applied voltage. Histograms were obtained from three independent replicas per system and condition, based on the time evolution of the distance R between the fluorophore attachment sites, assuming a fixed Förster radius at which energy transfer efficiency between donor and acceptor is 50%. Note that the fluorophores are not explicitly modeled in the MD simulations, and R_0 also depends on the relative orientation of the donor–acceptor pair. The reported distances therefore reflect only the positions of the attachment sites; nevertheless, they likely account for most of the differences observed experimentally.

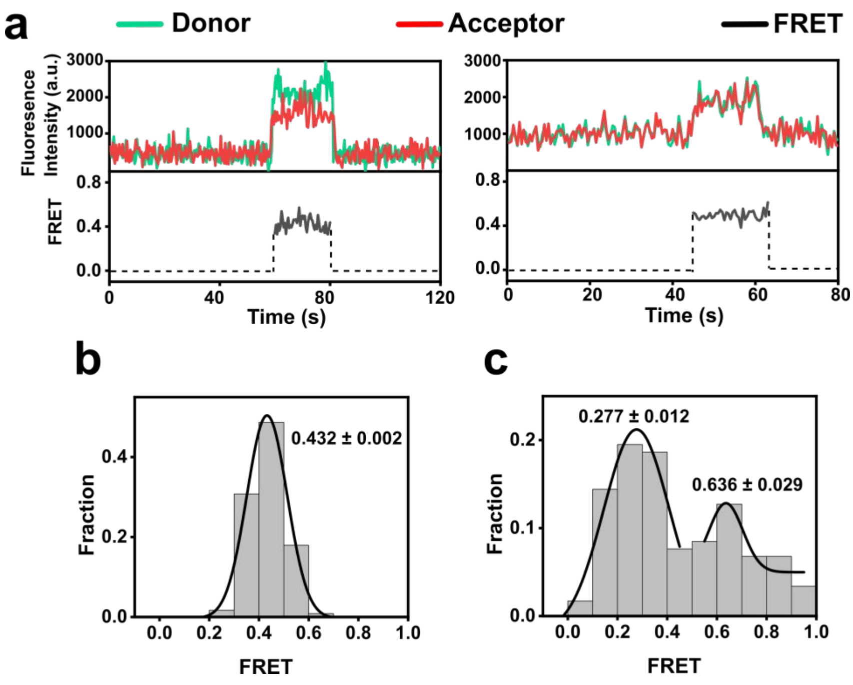

Figure S12. (a) Representative time traces of donor fluorescence (green), acceptor fluorescence (red), and FRET efficiency (black) illustrating only individual nucleosomes (without Chd1, ATPγS, or Mg^{2+}) confined in the nanocavity at 0 mV. Histograms of the mean FRET distributions for (b) in the absence of Chd1, ATPγS, or Mg^{2+} (N = 27) and (c) in the presence of Chd1, ATPγS, and Mg^{2+} (N = 38).

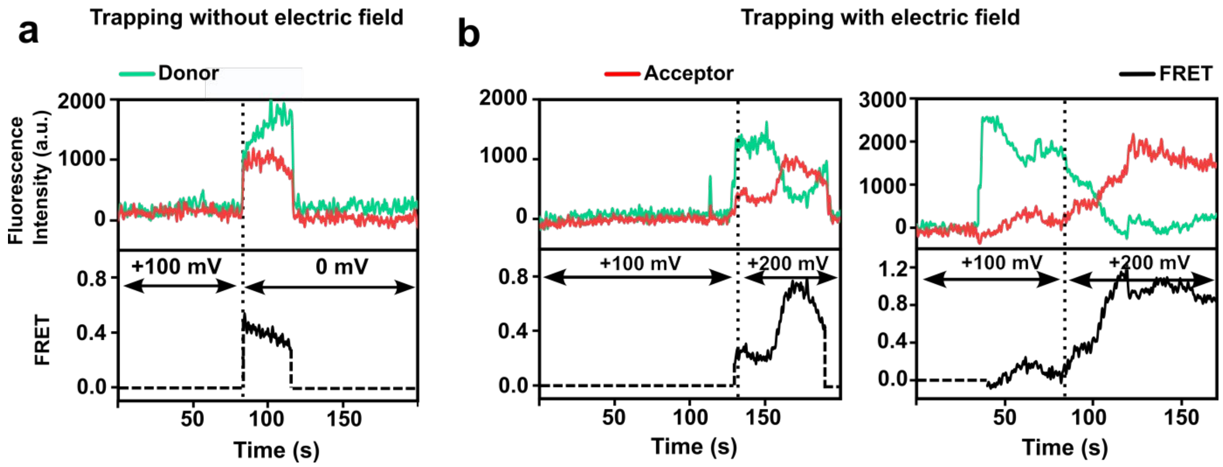

Figure S13. Weak interaction between two nucleosomes labeled with Cy3 and Cy5, respectively. Nucleosomes were loaded from pre-mixed solution. Representative time traces of donor fluorescence (green), acceptor fluorescence (red), and FRET efficiency (black) recorded (a) without electric field and (b) with electric field.

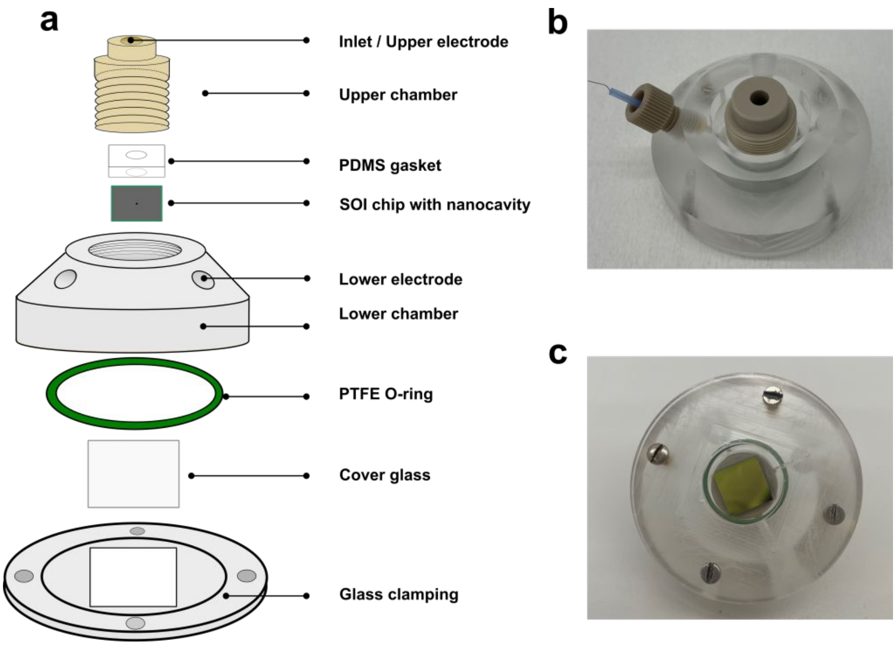

Figure S14. (a) Schematic of the custom-fabricated flow cell. (b) Top-view and (c) bottom-view optical images of the device.

Supplementary Method.

Molecular dynamics (MD) simulation.

All the MD runs were carried out using GROMACS 2024[3] with a time step $\Delta t = 2.0$ fs. The force field used is the same as that employed by Winogradoff & Aksimentiev,[4] based on Amber99sb-ILDN-PHI with bsc0 variant for DNA.[5] TIP3P model was used for water,[6] and non-bonded corrections were applied for NaCl[7] and charged groups (CUFIX).[8] A cutoff of 10 Å was used for the short-range nonbonded interactions. Particle mesh Ewald[9] method with a 1.6 Å spaced grid is used for long-range electrostatic interactions. A stochastic v-rescale thermostat[10] with a coupling constant of 0.1 ps applied to the entire system was used for all the simulations. Constraints were applied to bonded hydrogens using the SETTLE[11] algorithm for water and LINCS[12] for the other molecules. The initial velocities were generated from a Maxwell-Boltzmann distribution at 300 K. Periodic boundary conditions were applied in all three spatial dimensions. The membrane atoms were fixed in all directions and kept frozen in all the simulations. Production runs were performed at constant volume (NVT ensemble).

Membrane preparation. The membrane is made of uncharged hydrophilic dummy Lennard-Jones atoms (σ=0.37418 nm, ϵ=0.84 kJ/mol), having a simple cubic structure with atomic distance of 0.21 nm. A pore with a minor diameter of 6 nm is drilled through the membrane, using a smoothed function fitted from experimentally derived shape.

Nucleosome preparation. The complete structure of the histones, composing the nucleosome core protein, are taken from PDB 1KX5.[13] The dsDNA includes a 147-bp structure, wrapping the protein, elongated on the two sides with a shorter 19-bp or 3-bp and a longer 39-bp dsDNAs. The 147-bp structure is based on the Widom 601 sequence,[14] and its structure is taken from PDB 3LZ0.[15] The 19-bp, 3-bp and 39-bp dsDNAs are generated and merged with ChimeraX.[16] The center of mass of the protein core of the nucleosome is placed at an initial distance of 9 nm from the membrane upper surface.

Solvation and equilibration. The final system is solvated into a rectangular box of 22x22x34 nm^3 and the total charge is neutralized by ionizing the system at 0.15M with NaCl, using GROMACS *solvate* and *genion* tools. The solvated system is then minimized for 1000 steps *via* descent gradient and then equilibrated to the correct temperature with an NPT simulation until the system reached a steady state volume (~5 ns). The nucleosome atoms were initially restrained (1000 kJ/mol/nm^2) and progressively halving the constraints every 500 ps during the first 2 ns; then the complex was completely free. Pressure coupling was conducted using a Parrinello-Rahman barostat[17] in a semi-isotropic manner, with separate coupling for the x/y plane and the z-axis. The reference pressure was set to 1 bar with a compressibility of 4.5×10^{-5} bar^{-1}, and a coupling constant of 5.0 ps.

References

(1) Zeng, S.; Wen, C.; Solomon, P.; Zhang, S.-L.; Zhang, Z. Rectification of Protein Translocation in Truncated Pyramidal Nanopores. *Nat. Nanotechnol.* **2019**, *14* (11), 1056–1062. https://doi.org/10.1038/s41565-019-0549-0.

(2) Zeng, S.; Chinappi, M.; Cecconi, F.; Odijk, T.; Zhang, Z. DNA Compaction and Dynamic Observation in a Nanopore Gated Sub-Attoliter Silicon Nanocavity. *Nanoscale* **2022**, *14* (33), 12038–12047. https://doi.org/10.1039/D2NR02260E.

(3) Abraham, M. J.; Murtola, T.; Schulz, R.; Páll, S.; Smith, J. C.; Hess, B.; Lindahl, E. GROMACS: High Performance Molecular Simulations through Multi-Level Parallelism from Laptops to Supercomputers. *SoftwareX* **2015**, *1–2*, 19–25. https://doi.org/10.1016/j.softx.2015.06.001.

(4) Winogradoff, D.; Aksimentiev, A. Molecular Mechanism of Spontaneous Nucleosome Unraveling. *Journal of Molecular Biology* **2019**, *431* (2), 323–335. https://doi.org/10.1016/j.jmb.2018.11.013.

(5) Pérez, A.; Marchán, I.; Svozil, D.; Sponer, J.; Cheatham, T. E.; Laughton, C. A.; Orozco, M. Refinement of the AMBER Force Field for Nucleic Acids: Improving the Description of α/γ Conformers. *Biophysical Journal* **2007**, *92* (11), 3817–3829. https://doi.org/10.1529/biophysj.106.097782.

(6) Jorgensen, W. L.; Chandrasekhar, J.; Madura, J. D.; Impey, R. W.; Klein, M. L. Comparison of Simple Potential Functions for Simulating Liquid Water. *The Journal of Chemical Physics* **1983**, *79* (2), 926–935. https://doi.org/10.1063/1.445869.

(7) Joung, I. S.; Cheatham III, T. E. Determination of Alkali and Halide Monovalent Ion Parameters for Use in Explicitly Solvated Biomolecular Simulations. *The journal of physical chemistry B* **2008**, *112* (30), 9020–9041.

(8) Yoo, J.; Aksimentiev, A. New Tricks for Old Dogs: Improving the Accuracy of Biomolecular Force Fields by Pair-Specific Corrections to Non-Bonded Interactions. *Phys. Chem. Chem. Phys.* **2018**, *20* (13), 8432–8449. https://doi.org/10.1039/C7CP08185E.

(9) Darden, T.; York, D.; Pedersen, L. Particle Mesh Ewald: An $N\cdot\log(N)$ Method for Ewald Sums in Large Systems. *The Journal of Chemical Physics* **1993**, *98* (12), 10089–10092. https://doi.org/10.1063/1.464397.

(10) Bussi, G.; Donadio, D.; Parrinello, M. Canonical Sampling through Velocity Rescaling. *The Journal of Chemical Physics* **2007**, *126* (1), 014101. https://doi.org/10.1063/1.2408420.

(11) Miyamoto, S.; Kollman, P. A. Settle: An Analytical Version of the SHAKE and RATTLE Algorithm for Rigid Water Models. *Journal of computational chemistry* **1992**, *13* (8), 952–962.

(12) Hess, B.; Bekker, H.; Berendsen, H. J.; Fraaije, J. G. LINCS: A Linear Constraint Solver for Molecular Simulations. *Journal of computational chemistry* **1997**, *18* (12), 1463–1472.

(13) Davey, C. A.; Sargent, D. F.; Luger, K.; Maeder, A. W.; Richmond, T. J. Solvent Mediated Interactions in the Structure of the Nucleosome Core Particle at 1.9 Å Resolution. *Journal of molecular biology* **2002**, *319* (5), 1097–1113.

(14) Lowary, P.; Widom, J. New DNA Sequence Rules for High Affinity Binding to Histone Octamer and Sequence-Directed Nucleosome Positioning. *Journal of molecular biology* **1998**, *276* (1), 19–42.
(15) Vasudevan, D.; Chua, E. Y.; Davey, C. A. Crystal Structures of Nucleosome Core Particles Containing the '601'Strong Positioning Sequence. *Journal of molecular biology* **2010**, *403* (1), 1–10.
(16) Pettersen, E. F.; Goddard, T. D.; Huang, C. C.; Meng, E. C.; Couch, G. S.; Croll, T. I.; Morris, J. H.; Ferrin, T. E. UCSF ChimeraX: Structure Visualization for Researchers, Educators, and Developers. *Protein science* **2021**, *30* (1), 70–82.
(17) Parrinello, M.; Rahman, A. Polymorphic Transitions in Single Crystals: A New Molecular Dynamics Method. *Journal of Applied physics* **1981**, *52* (12), 7182–7190.
